# Abcès mésentérique du au *Chryseobacterium meningosepticum* révélant la maladie de Castleman à localisation mésentérique chez un jeune adulte immunocompetent

**DOI:** 10.11604/pamj.2022.41.99.19121

**Published:** 2022-02-04

**Authors:** Manix Ilunga Banza, Nathalie Dinganga Kapessa, Néron Tapenge Shutsha, Pius Wonga Omole, Yannick Tietie Ben N’dwala, Trésor Kibangula Kasanga, Dimitri Kanyanda Nafatalewa, Prince Muteba Katambwa

**Affiliations:** 1 Département de Chirurgie des Cliniques universitaires de Lubumbashi, Université de Lubumbashi, Haut Katanga, Lubumbashi République Démocratique du Congo

**Keywords:** Abcès mésentérique, maladie de Castleman, immunocompétent, cas clinique, Mesenteric abscess, chryseobacterium meningosepticum, Castleman disease, immunocompetent, case report

## Abstract

Un abcès mésentérique causé par le Chryseobacterium meningosepticum est une entité clinique extrêmement rare, le plus souvent retrouvé sur un terrain d´immunodéficience et la maladie de Castleman peut être révélée par un abcès mésentérique. Nous présentons le cas d´un patient de 23 ans admis pour péritonite aiguë généralisée évoluant depuis 2 semaines. L´échographie abdominale montrait une masse hypoéchogène dans les anses grêles sans donner des plus amples détails. A la laparotomie, du pus avait été retrouvé dans la grande cavité et un abcès mésentérique présent dans le mésentère jéjunal à 35 cm de l´angle de Treitz sans aucune ouverture de l´anse en regard mais également une adénopathie mésentérique en regard de l´abcès. La pyoculture a isolé le Chryseobacterium meningosepticum et l´adénopathie mésentérique enlevée a montré à l´analyse histologique des anomalies structurales évoquant le type vascularisation hyalinisée de la maladie de Castleman. Le traitement a consisté en une incision et drainage de l´abcès avec résection de la coque et un nettoyage de la cavité abdominale au sérum physiologique. La Ciprofloxacine était le seul antibiotique sensible au Chryseobacterium meningosepticum. Les suites post-opératoires étaient simples avec sortie du patient au 10^e^ jour post-opératoire. Un suivi clinique et paraclinique du patient pendant 12 mois n´a objectivé aucune autre adénopathie ni aucune récidive. Le but de cette publication est de présenter un cas extrêmement rare associant un abcès mésentérique à Chryseobacterium meningosepticum et une maladie de Castleman unicentrique à localisation mésentérique chez un immunocompétent ainsi que les modalités de cette prise en charge.

## Abstract (en)

Mesenteric abscess caused by chryseobacterium meningosepticum is an extremely rare clinical entity, most often found in immunodeficient patients. Castleman disease can manifest as a mesenteric abscess. We here report the case of a 23-year-old patient admitted with generalized acute peritonitis evolving over the last 2 weeks. Abdominal ultrasound showed hypoechoic mass in the intestinal loops, without any further details. During laparotomy, pus was found in the large cavity, a mesenteric abscess was detected in the mesentery of the jejunum 35 cm from the angle of Treitz, without loop perforation and mesenteric adenopathy was found in corresponence with the abscess. Pyoculture isolated chryseobacterium meningosepticum. Histological analysis of mesenteric adenopathy biopsies showed structural anomalies, suggesting hyaline vascular type of Castleman disease. Treatment was based on incision and drainage of the abscess, with pyogenic membrane removal and abdominal cavity lavage with physiological saline solution. Chryseobacterium meningosepticum was only susceptible to Ciprofloxacin. The postoperative course was simple and the patient was discharged on postoperative day 10 after surgery. Clinical and paraclinical follow-up at 12 months did not show any other lymphadenopathy or recurrence. The purpose of this study is to show an extremely rare case of mesenteric abscess due to chryseobacterium meningosepticum associated with isolated mesenteric Castleman disease in an immunocompetent patient and management approaches.

## Introduction

L´abcès mésentérique est surtout décrit chez des patients avec un terrain particulier tel qu´une immunodépression à VIH [1], un traitement aux immunosuppresseurs [2], une maladie de Crohn [3]. Les germes les plus souvent retrouvés à l´origine des abcès mésentériques sont le Bacille de koch [4], le complexe mycobacterium avium [1], le yersinia enterocolitica [2]. La maladie de Castleman n´est pas décrite comme un terrain particulier pour la survenue des abcès mésentériques. Cette maladie dont le nom est attribué à Benjamin Castleman a été décrite pour la première fois par cet anatomopathologiste américain en 1954 [5] et identifiée comme une entité nosologique 2 ans plus tard [6]. Il s´agit d´une affection relativement rare caractérisé par une hyperplasie lymphoïde dont le siège électif est le médiastin. C´est une pathologie qui peut se développer là où les ganglions lymphatiques existent, surtout au dépens des chaines ganglionnaires du médiastin avec une fréquence élevée le long de l´arbre trachéo-bronchique et des hiles pulmonaires. L´étiopathogénie de cette affection reste inconnue, quoique plusieurs hypothèses ont été avancées, essentiellement en cas de maladie de Castleman multicentrique, impliquant des phénomènes inflammatoires chroniques avec dérèglement de la production d´interleukine 6 [7], l´immunodépression à VIH, et enfin une réponse immunitaire atypique à l´infection par le Human Herpes Virus 8 ( HHV8) [8].

Très peu d´articles rapportent le *Chryseobacterium meningosepticum* comme cause d´abcès mésentérique. C´est une bactérie gram négatif qui cause préférentiellement la maladie chez les nouveau-nés prématurés et les enfants [9], saprophyte d´origine hydrique [10], agent pathogène opportuniste d´une faible virulence qui cause rarement des ´infections graves chez les adultes. Les cellulites représentent 3% de toutes les infections dues au *Chryseobacterium meningosepticum* décrites [11]. D´autres lésions causées par ce germe chez les adultes sont décrites notamment une arthrite septique du coude sur prothèse [10], cellulite et sepsis chez une femme avec *pemphigus vulgaris* [12], une septicémie et péritonite [13], une septicémie avec hématome rétropéritonéale et effusion pleurale [14]. Un abcès mésentérique peut cacher une tumeur abdominale maligne [15] qu´il convient impérativement de rechercher; mais la maladie de Castleman n´est pas une tumeur maligne. Elle se présente cliniquement sous deux formes à savoir la forme uni-centrique correspondant à une tumeur unique pauci ou asymptomatique, d´origine lymphatique dont on distingue trois types histologiques (type à vascularisation hyalinisée représente 91% des cas de l´adulte, type plasmocytaire et type mixte) et la forme multicentrique qui touche plusieurs territoires ganglionnaires [16]. Ses localisations abdominales et pelviennes sont rares: intrapéritonéales dans 5,7% des cas (dont 3,5% dans le mésentère), et rétro-péritonéales dans 6,6% des cas [17].

Quelque soit son étiologie, l´abcès mésentérique doit être évacué soit par laparotomie soit par laparoscopie. Ensuite un traitement médical selon le germe trouvé et l´antibiogramme est conduit pour une meilleure prise en charge; l´exérèse chirurgicale est recommandée en première intention, complète et assure généralement la guérison dans la maladie de Castleman [16]. Cependant nous n´avons pas trouvé dans la littérature des cas d´abcès mésentériques causés par le *Chryseobacterium meningosepticum* associée à la maladie de Castleman; ceci a donc été le motif de notre travail dans le but de faire savoir qu´un abcès mésentérique à *Chryseobacterium meningosepticum* peut révéler une maladie de Castleman chez un patient immunocompétent.

## Patient et observation

**Présentation du patient**: il s´agit d´un patient âgé de 23 ans, venu consulter aux cliniques universitaires de Lubumbashi en date du 27 janvier 2019 pour douleur abdominale. L´histoire de la maladie remontait à 2 semaines de notre consultation par la survenue brutale d´une douleur abdominale pour laquelle le patient s´était automédiquée aux antalgiques usuels faits de paracétamol comprimé 500mg et de papaverine comprimé sans succès pendant 1 semaine. La persistance de la douleur et la survenue de la fièvre avait motivé le patient à consulter un centre de santé dans la ville de Kolwezi située à 300Km de notre ville de Lubumbashi où des perfusions lui ont été administrées ainsi que des antipyrétiques. Une décision d´intervention chirurgicale y avait été prise mais le patient n´y avait pas adhérer. Sur demande de la famille, le patient avait ainsi été amené aux Cliniques Universitaires de Lubumbashi pour une meilleure prise en charge. A notre anamnèse, le patient s´est plaint d´une vive douleur abdominale et d´une fièvre. Aucun antécédent médico-chirurgical, ni toxico-allergique particulier n´avait été note. Au complément d´anamnèse, le patient avait signalé une douleur abdominale de survenue brutale devenant de plus en plus intense, permanente, localisée d´abord dans l´hypochondre droit et flanc droit puis s´est généralisée, insomniante, exacerbée par l´inspiration profonde, atténuée légèrement par l´antéflexion du tronc. Une fièvre sans horaire, pas d´arrêt de matières et de gaz, pas de vomissements.

**Résultats cliniques**: à l´examen physique, l´état général était marqué par une fièvre à 38,6 dégré celcius et un faciès souffrant. Les conjonctives palpébrales étaient colorées, les bulbaires anictériques, la bouche était propre et la langue était sèche, aucune adénopathie n´avait été retrouvée à la palpation. Le thorax était polypnéique à 30 cycles par minutes, cœur tachycarde à 105 battements par minutes. L´abdomen était légèrement ballonné, la respiration abdominale présente, l´ombilic n´était pas déplissé. La palpation superficielle ne décelait pas une hyperesthésie cutanée. La douleur abdominale était généralisée mais maximale dans la région de l´hypochondre droit et le flanc droit avec une contracture généralisée. Le cri ombilical était présent. Nous avions noté une matité déclive mobilisable. La matité préhépatique était conservée. A l´auscultation, avions noté la présence des bruits hydro-aériques. Au toucher rectal, le sphincter était tonique, ampoule rectale vide, le cul de sac de Douglas bombé et sensible. Un diagnostic clinique de péritonite aiguë généralisée avait été retenu compliquée de déshydratation au plan B mais avions suspecter une perforation de la vésicule biliaire.

**Figure 1:**
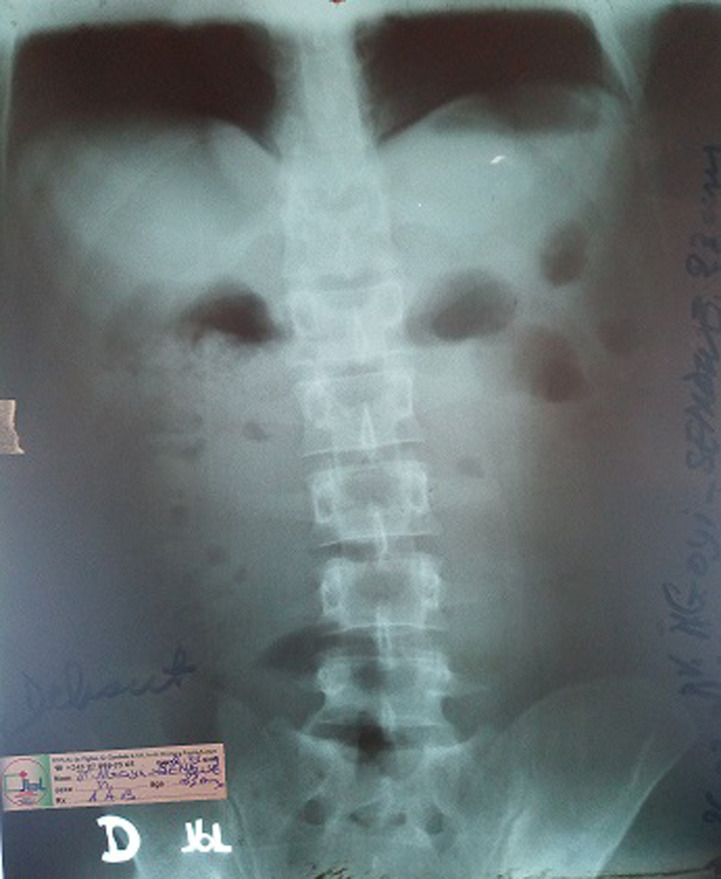
radiographie abdomen à blanc

**Démarche diagnostique**: nous avions ainsi demandé des examens paracliniques faits de: 1) la radiographie abdomen à blanc patient débout avec visualisation des hémicoupoles diaphragmatiques qui a révélée l´absence de pneumopéritoine (pas de croissant gazeux sous diaphragmatique) mais la présence de quelques niveaux hydro-aériques suggestifs d´iléus sans évidence d´occlusion mécanique (Figure 1). 2) L´échographie abdominopelvienne avait montré un foie et une vésicule biliaire intacte, un pancréas et une rate normale, des reins normaux. Une masse intra-abdominale hypoéchogène avait été décelée à l´échographie mais sans de plus amples détails; elle avait noté également la présence d´un épanchement à liquide trouble, plus localisé dans le pelvis, une sensibilité abdominale à l´échopalpation: un diagnostic de péritonite avec iléus reflexe avait donc été retenue à l´échographie. 3) Les résultats de laboratoire avant l´intervention chirurgicale que voici: a) bactériologie: Widal TH(-) TO(-); sérologie HIV: négative. b) Biochimie: glycémie 95 ml/dl; CRP: 8,2 mg%; urée 21mg/dl; créatinine 1,02mg/dl; sodium 138mEq/l; potassium 4,0mEq/l; calcium 9,3mg/dl. d) Hématologie: erythrocytes 448000; hématocrite 35%; kémoglobine 14,8g/dl; vitesse de sédimentation 53mm/h; plaquette 255000/mm^3^, globules blancs 6890/mm^3^, volume globulaire Moyenne 79,2µm^3^, formule leucocytaire granulocytes (65%) lymphocytaire (27%) monocytes (8%). e) Parasitologie: examen des selles à frais négatif, goutte épaisse négative (0 parasites/µl). f) Temps de saignement: 2´ 30´´; temps de coagulation: 4´00´´; groupe sanguin et rhésus 0+. Ainsi, après examens cliniques et paracliniques une péritonite aiguë généralisée avait retenue comme diagnostic sans évidence de sa cause. Une décision d´opérer par une laparotomie exploratrice avait été prise après une évaluation anesthésique.

**Figure 2:**
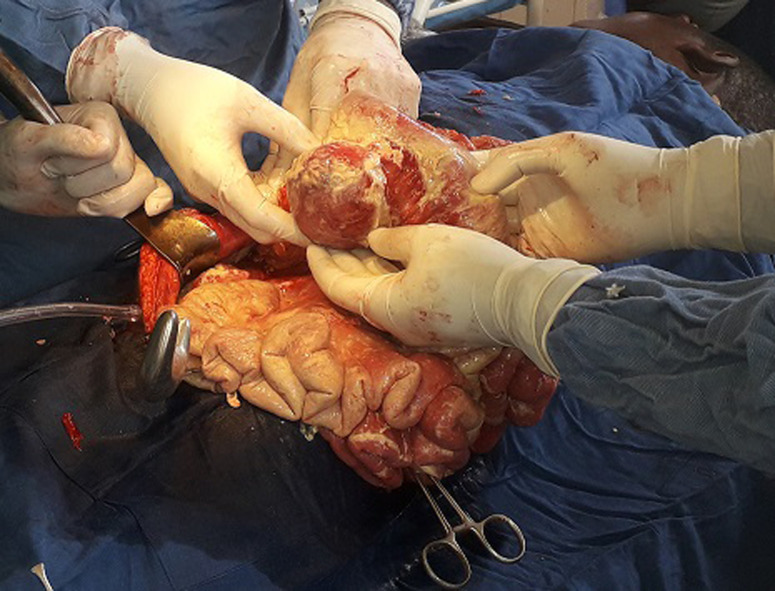
abcès mésentérique visible

**Figure 3:**
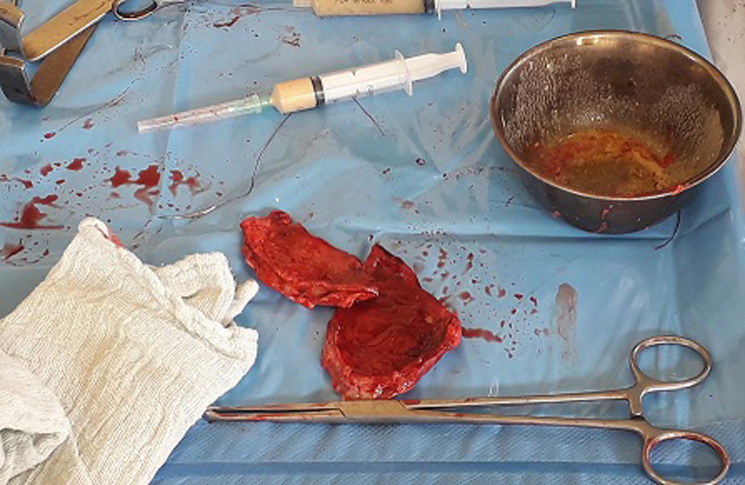
la coque réséquée pour prélèvement biopsique et seringues contenant du pus prélevé pour l’anatomopathologie

**Figure 4:**
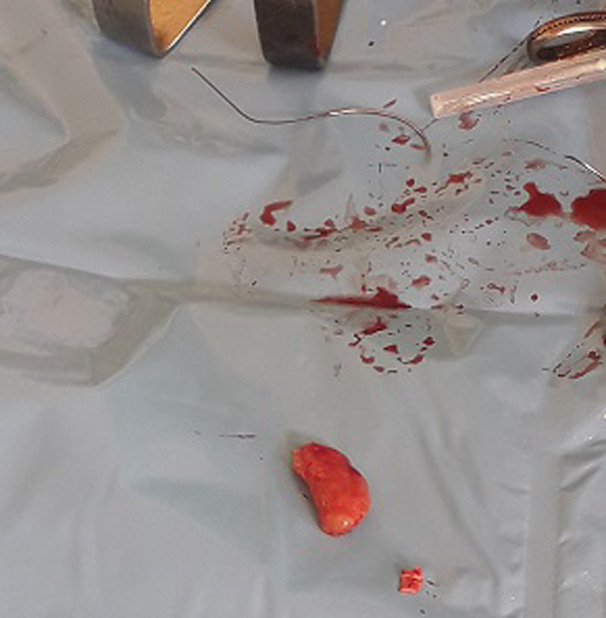
ganglion prélevé pour l’anatomopathologie

**Intervention thérapeutique**: notre patient était classé ASA III, et une réanimation pendant 8 heures avait été instaurée. Elle a consisté en: la pose d´une sonde nasogastrique numéro 14; la pose d´une sonde vésicale à demeure numéro 16; la pose d´une voie veineuse centrale avec une réanimation hydrique selon la formule de Lewis repartie en sérum physiologique 1 litre pendant 3 heures, Haemacèle 1 litre pendant 2 heures et Ringer Lactate 1 litre pendant 3 heures. Une antibiobioprophylaxie au Ceftriaxone avait été instaurée 2 grammes avant l´intervention chirurgicale. La laparotomie médiane sus et sous-ombilicale a été la voie d´abord. A l´entrée dans la cavité abdominale, pas de pneumopéritoine et pas d´odeur particulière. Un épanchement trouble et purulent d´odeur fade dans la grande cavité abdominale était noté, aspiré grâce à une canule d´aspiration et quantifié à 350 cc. Il y´avait la présence d´une quantité importante de fibrines disséminée dans la cavité abdominale recouvrant les anses grêles, le colon, le foie. L´exploration abdominale a mis en évidence une masse ovoïde localisé dans le mésentère jéjunale à 25 cm de l´angle de Treitz, encapsulée, rénitente, mesurant 15 x 10cm, l´anse grêle en regard était intact, de coloration rosé, perméable (Figure 2). A côté de la masse mésentérique, il y avait la présence d´une seule adénopathie mésentérique, ovoïde de 2,5 x 1,5 cm. Avant l´ouverture de la masse, une première seringue stérile a servi pour prélever le liquide purulent de la cavité abdominale et une deuxième seringue stérile avait été introduite par le centre de la masse et a ramené du pus franc, jaunâtre et bien lié qui ont tout de suite été amenée au laboratoire pour des analyses (Figure 3). La prise en charge chirurgicale a consisté à l´ouverture de cette masse mésentérique par une petite incision cruciforme de 0,5cm en son centre élargie d´abord par une pince droite, ce qui a ramené 120 cc de pus jaunâtre bien lié, fade recueilli dans un godet stérile. Ensuite à l´aide de l´index introduit dans la cavité contenant du pus pour rompre des possibles cloisons. Nous avions ensuite élargie notre incision au ciseau et avions réséqué toute la coque de façon circonférentielle qui a été mis dans un récipient contenant du sérum physiologique et tout de suite amené au service d´anatomopathologie pour une biopsie de cette membrane (Figure 3). Ensuite cette cavité dont la coque a été entièrement réséquée a été laissé ouverte en contact avec le péritoine. Une hémostase rigoureuse contournant les berges de la cavité réséquée a été faite en surjet au fil à résorption lente Vicryl numéro 2/0. L´anse jéjunale en regard n´a pas été lésée ni privée de sa vascularisation. L´adénopathie mésentérique unique se trouvant dans le mésentère jéjunal en regard de cette masse avait également été prélevé en entièreté pour les examens anatomopathologiques (Figure 4). La vérification du mésentère n´a révélé aucune autre adénopathie. Nous avions ensuite nettoyé abondamment la cavité abdominale avec 5 litres de sérum physiologique tiède en insistant sur les loges sous phréniques, les gouttières pariéto-coliques et le douglas. Deux drains lamellaires avaient été laissés en place dont l´un dans le douglas extériorisé dans la fosse iliaque gauche et l´autre en regard de la cavité qui contenait du pus extériorisé par le flanc droit. La cavité abdominale a été ensuite refermée en deux plans au fil à résorption lente Vicryl. Le patient avait d´abord était gardé en réanimation où il avait reçu une unité de sang 450cc isogroupe et isorhésus en post-opératoire immédiat et gardé en réanimation pendant 48 heures. Une antibiothérapie probabiliste fait de Céfotaxime 1 gramme toutes les 8 heures en Intraveineuse directe associée au Métronidazole infusion 1 infusion toutes les 8 heures jusqu´à la réception des résultats de la pyoculture.

**Suivi et résultats des interventions thérapeutiques**: le retour de transit était effectif après 48 heures et le patient avait ainsi été ramené en salle d´hospitalisation et une diète liquide fait de thé chaud a été instauré mais la fièvre avait persisté jusqu´au 3^e^ jour post-opératoire. Le premier pansement a été réalisé au 3^e^jour post-opératoire montrant un pansement médian propre et sec et un pansement aux drains teinté de sécrétions séro-hématiques. Une douleur abdominale péri-lésionnelle. Les drains lamellaires ont ainsi été retiré au 3^e^ jour post-opératoire car na ramenant plus aucune sécrétion. La persistance de la fièvre jusqu´au 4^e^ jour post-opératoire a fait réaliser un nouveau prélèvement de sang pour une hémoculture et une goutte épaisse. Les résultats obtenus le lendemain du prélèvement était négatif pour la malaria et après 4 jours d´hémoculture était également négatif, aucun germe retrouvé. Les résultats de la pyoculture (faite sur gélose chocolatée) reçu le 4^e^ jour post-opératoire sur les deux échantillons ont isolé le même germe nommé *Chryseobacterium meningosepticum* et l´antibiogramme a donné comme résultat une sensibilité seulement à la ciprofloxacine et une résistance à plusieurs antibiotiques usuels notamment la Ceftriaxone, la Céfotaxime, la Ceftazidine, la gentamycine, l´imipenème, amoxicilline et la tétracycline. Ainsi au 4^e^ jour post-opératoire, la Ceftriaxone a été retirée et remplacée par la Ciprofloxacine infusion 500mg à raison de 3x500mg/j. Et au 5^e^ jour post-opératoire, nous avons noté une disparition de la fièvre. Un état général du malade nettement bon avec une nette disparition de la douleur abdominale. Le second pansement a été réalisé au 7^e^ jour post-opératoire. L´examen de l´abdomen au 7^e^ jour post-opératoire a montré un abdomen non ballonné, recouvert d´un pansement médian sec et propre, dont l´ablation du pansement montre une plaie incisionnelle médiane en voie de cicatrisation, un abdomen souple, dépressible, non sensible. Le bilan paraclinique de contrôle réalisé au 7^e^ jour post-opératoire a montré les résultats suivants:

**Hématologie**: érythrocytes: 440000; h ématocrite: 35%; hémoglobine: 12,8g%; vitesse de sédimentation: 39mm à la 1^e^^re^ heure; plaquettes sanguines: 297000/mm^3^; globules blancs: 6710/mm^3^; volume globulaire moyen: 78,7m^3^; concentration corpusculaire moyenne en hémoglobine: 34,1%; formule leucocytaire: granulocytes (77%); lymphocytes (11%); monocytes (12%); éosinophile (0%); hémoculture et antibiogramme: négative; sérologie HIV: négative.

**Biochimie**: CRP 2,4 mg% glycémie à jeun 105 mg%.

**Bactériologie**: urine (examen cytobactériologique et coloration gram) absence.

Le malade a été libéré de l´hôpital au 10^e^jour post-opératoire après une ablation complète des fils de suture sans aucun signe d´infection locale de la plaie opératoire. Les analyses anatomopathologiques avaient trouvé comme résultats: a) le tissu prélevé a montré un tissu fibreux contenant une cavité vide faisant état d´une membrane d´abcès; b) le ganglion présente une hyperplasie lymphoïde avec multiples follicules germinaux disposés de façon concentrique réalisant une image en bulbe d´oignon autour d´axes vasculaires à parois hyalinisées. Le Stroma étant le siège de nombreux vaisseaux à paroi hyaline et des lymphocytes mature; l´aspect histologique est celui de la maladie de Castleman dans sa forme vasculaire hyalinisée.

Le patient a ainsi été suivi pendant 12 mois à la recherche d´une quelconque adénopathie qui pourrait traduire une forme multicentrique de la maladie de Castleman ou une récidive mésentérique de la maladie de Castleman. Cliniquement, aucun ganglion n´a été détecté durant ce temps de suivi. Deux radiographies thoraciques et deux échographies abdominales ont été réalisées à intervalle de 1 mois à la recherche d´une adénopathie profonde qui apparaitrait dans les 3 mois mais les examens réalisés étaient tous négatifs. Aucune récidive de la maladie de Castleman n´a été trouvée sur un suivi de 12 mois.

**Consentement éclairé**: après des explications claires et détaillées, le patient a donné personnellement par écrit son consentement éclairé vu qu´il est majeur pour la réalisation de ce travail.

## Discussion

Un abcès mésentérique est une entité assez rare en chirurgie digestive; il est surtout l´apanage des patients immunodéprimés dont l´étiologie est dominée par les germes opportunistes comme le bacille de koch, le complexe *Mycobacterium avium* [1,4] quoi que d´autres germes soient retrouvés chez les immunocompétents tels que le Yersinia enterolitica [2], parfois même aucun germe n´est retrouvé dans cet abcès si celui-ci est du à une tumeur maligne [15]. Chez notre patient, immunocompétent chez qui une maladie de Castleman uni-centrique à localisation mésentérique a été retrouvée de façon fortuite, l´abcès mésentérique était causé par un germe rare, le *Chryseobacterium meningosepticum*. Ce dernier est normalement reconnu comme étant à l´origine des méningites chez les prématurés et chez les nouveau-nés ainsi que de pneumonie, d´endocardite, de bactériémie post-opératoire et parfois de méningite chez les adultes [18] avec une grave maladie sous-jacente telle que une cirrhose de foie, une aplasie médullaire, une insuffisance cardiaque ou rénale; il peut être isolé dans le sang, le liquide céphalo-rachidien ou sur les plaies de brulure [4] tandis que le pus prélevé dans la cavité abdominale et dans l´abcès mésentérique a été notre source d´isolement de ce germe.

Les premiers cas d´infection abdominale à *Chryseobacterium meningosepticum* ont été décrits comme infections nosocomiales mais également le premier patient décrit avec abcès abdominal à ce germe [19] par contre notre patient n´a pas contracté son infection à *Chryseobacterium meningosepticum* à l´hôpital, ce n´est donc pas dans notre cas une infection nosocomiale telle que décrit dans diverses littératures. La maladie de Castleman localisé demeurent longtemps asymptomatique, souvent de découverte fortuite [16] justifiant également sa découverte fortuite dans notre observation clinique . Les patients avec abcès mésentériques peuvent présenter un tableau d´abdomen aigu chirurgical, caractérisée par la douleur abdominale (maître symptôme), qu´il s´agisse d´une occlusion intestinale par compression de la masse sur l´intestin grêle [1], d´une péritonite [3,4], d´une urétéro-hydronéphrose secondaire à une compression de l´urétère par la masse mésentérique [3] mais aussi la fièvre traduisant un tableau de suppuration profonde qu´il faille impérativement évacué. Et donc la douleur abdominale et la fièvre sont des signes quasi-constants expliquant ainsi la symptomatologie douloureuse et fébrile de notre patient. La localisation de l´abcès mésentérique peut être au niveau du méso de l´intestin grêle, notamment sur l´iléon terminale et la région iléo-cœcale [2,3,15], expliquant ainsi celle de notre patient au niveau dans la portion jéjunale du mésentère à 25 cm de l´angle de Treitz. Aucune atteinte de l´anse jéjunale n´avait été notée, aucune ouverture intestinale, aucune tumeur du tube digestif notée mais la présence d´une adénopathie en regard de la masse mésentérique. Cette adénopathie prélevée nous a permis de poser de façon fortuite le diagnostic de la maladie de Castleman à localisation mésentérique, tout en sachant qu´une soixantaine de cas de maladie de Castleman à localisation abdominale a été décrite, essentiellement rétropéritonéale et environ 20 cas mésentériques [17].

Quelque soit la cause de l´abcès mésentérique, le traitement chirurgical s´impose. Il consiste en un drainage du contenu de l´abcès soit par laparotomie [1,2] soit un drainage percutané [3], le prélèvement du pus pour l´analyse bactériologique et l´isolement du germe en vue d´un traitement adéquat. L´exérèse chirurgicale reste dans la majorité des cas le meilleur moyen pour assurer le diagnostic positif et permettre un geste curatif dans la maladie de Castleman; l´exérèse radicale est la règle quoique difficile en cas de masse intimement adhérent aux structures voisines [16]. Ce qui justifie de ce fait notre prise en charge chirurgicale des 2 pathologies conjointes retrouvées chez notre patient à savoir abcès mésentérique et maladie de Castleman.

L´antibiogramme n´a trouvé une sensibilité du germe uniquement à la ciprofloxacine. Et Ceci rejoint les diverses littératures qui attestent que la particularité du *Chryseobacterium meningosepticum* est d´être résistant à plusieurs antibiotiques souvent utilisés pour les autres bactéries gram négative [4,10]. Ce germe est reconnu comme étant résistant à plusieurs antibiotiques. Dans notre cas, sur 8 antibiotiques testés au laboratoire, seule la ciprofloxacine était sensible au *Chryseobacterium meningosepticum* alors que les 7 autres étaient résistants; il s´agissait de la Céfotaxime, la Ceftazidine, le Ceftriaxone, l´amoxicilline, la gentamycine, l´imipenème et la tétracycline. Une étude réalisée par BOBOSI Serengbe [20] en République Centrafricaine a trouvé que le *Chryseobacterium meningosepticum* était résistant à plusieurs antibiotiques tel que l´Amoxicilline, les céphalosporines de troisième génération tel que la Céfotaxime, la Ceftazidine, Ceftriaxone, Cefpirome), aux principaux aminosides (Gentamycine, Tobramycine, Amikacine), au chloramphénicol, à l´érythromycine, à la fosfomycine, à l´Azthreonam et à l´Imipenème tandis qu´il demeurait sensible à la pipéracilline seule ou associée au Tazobactam, à la Ciprofloxacine, à l´association Trimethoprime/Sulfamethoxazole, à la Rifampicine et à la Vancomycine. Ceci corrobore avec notre observation caractérisée par la persistance de la fièvre sous l´association Céfotaxime-métronidazole les 4 premiers jours post-opératoire avant les résultats de la pyoculture et l´isolement du germe. Le retrait de la Ceftriaxone et son remplacement par la Ciprofloxacine s´est spectaculairement suivie d´une chute de la fièvre dès le cinquième jour post-opératoire et d´une nette amélioration de l´état clinique du patient et même des examens paracliniques de contrôle.

Malgré la sortie de notre patient de l´hôpital au 10^e^jour post-opératoire sans aucune infection ni sur le site opératoire ni générale, il sied de signaler que l´infection par Chryseobacterium meningosepticum reste mortelle comme rapportés dans diverses littératures [9,10,20]. Le suivi clinique et paraclinique de notre patient pendant les 12 mois après intervention chirurgicale chez qui aucune récidive de la maladie de Castleman n´a été trouvée corrobore avec les diverses littératures de la quasi absence de récidive de cette maladie de Castleman dans sa forme monocentrique après exérèse chirurgicale du ganglion.

## Conclusion

Un abcès mésentérique peut être causé par le *Chryseobacterium meningosepticum*, germe aérobique, gram négatif. Mais sa coexistence avec la maladie de Castleman unicentrique à localisation mésentérique dans sa forme vasculaire hyalinisée existe quoi que non encore décrit et ce cas clinique présenté en est bien une illustration. Les deux entités sont rares chez un sujet immunocompétent mais possible, toutes relevant d´un traitement chirurgical absolu.

## References

[1] Mohar SM, Saeed S, Ramcharan A, Depaz H (2017). Small bowel obstruction due to mesenteric abscess caused by Mycobacterium avium complex in an HIV patient: a case report and literature review. J Surg Case Rep.

[2] Watanabe K, Watanabe N, Jin M, Matsuhashi T, Koizumi S, Onochi K (2014). Mesenteric lymph node abscess due to Yersinia enterocolitica: case report and review of the literature. Clin J Gastroenterol.

[3] Teeples TJ, Tabibian JH (2013). Images in clinical medicine: mesenteric abscess in Crohn´s disease. N Engl J Med.

[4] Cheung HYS, Siu WT, Yau KK, Ku CF, Li MKW (2005). Acute abdomen: an unusual case of ruptured tuberculous mesenteric abscess. Surg Infect (Larchmt).

[5] Castleman B, Towne VW (1954). Case records of the Massachusetts General Hospital: Case No 40231. N Engl J Med.

[6] Keller AR, Hochholzer L, Castleman B (1972). Hyaline-vascular and plasma-cell types of giant lymph node hyperplasia of the mediastinum and other locations. Cancer.

[7] Brandt SJ, Bodine DM, Dunbar CE, Nienhuis AW (1990). Dysregulated interleukin 6 expression produces a syndrome resembling Castleman´s disease in mice. J Clin Invest. aoû.

[8] Weisenburger DD, DeGowin RL, Gibson P, Armitage JO (1979). Remission of giant lymph node hyperplasia with anemia after radiotherapy. Cancer. aoû.

[9] Lin P-Y, Chu C, Su L-H, Huang C-T, Chang W-Y, Chiu C-H (2004). Clinical and microbiological analysis of bloodstream infections caused by Chryseobacterium meningosepticum in nonneonatal patients. J Clin Microbiol.

[10] Kumar R, Stephens JL (2004). Septic arthritis caused by *Chryseobacterium meningosepticum* in an elbow joint prosthesis. South Med J.

[11] Tuon FF, Campos L, Duboc de Almeida G, Gryschek RC (2007). Chryseobacterium meningosepticum as a cause of cellulitis and sepsis in an immunocompetent patient. J Med Microbiol. août.

[12] Sood S, Nerurkar V, Malvankar S (2010). Chryseobacterium meningosepticum cellulitis and sepsis in an adult female with Pemphigus vulgaris. Indian J Med Microbiol.

[13] Marnejon T, Watanakunakorn C (1992). Flavobacterium meningo-septicum septicemia and peritonitis complicating CAPD. Clin Nephrol.

[14] Lee S-W, Tsai C-A, Lee B-J (2008). Chryseobacterium meningosepticum sepsis complicated with retroperitoneal hematoma and pleural effusion in a diabetic patient. J Chin Med Assoc.

[15] Yamagata Y, Ando Y, Matsusaka K, Karube H, Onoyama H, Aikou S (2014). Poorly differentiated mesenteric carcinoma of unknown primary site detected by abscess formation: case report. World J Surg Oncol.

[16] Eloueriachi F, Caidi M, Zouaidia F, Ouchen F, Maidi M, Fennane H (2012). Castleman disease: unusual location of the chest. Pan Afr Med J.

[17] Capdevielle P, Lapprand M, Derrien JP, Courbil J, Courtois D, Barabe P (1983). Abdominal forms of Castleman´s disease. Gastroenterol Clin Biol.

[18] Padmaja P, Verghese S, Bhirmanandham CV, Ajith null, Thirugnanasambandham S, Ramesh S (2006). Chryseobacterium meningosepticum: an uncommon pathogen causing adult bacterial meningitis. Indian J Pathol Microbiol.

[19] Pokrywka M, Viazanko K, Medvick J, Knabe S, McCool S, William Pasculle A (1993). A flavobacterium meningosepticum outbreak among intensive care patients. American Journal of Infection Control.

[20] Bobossi-Serengbe G, Gody JC, Beyam NE, Bercion R (2006). First documented case of *Chryseobacterium meningosepticum meningitis*in Central African Republic. Med Trop (Mars).

